# Supplementary material for: Blockchain enabled privacy provisioning scheme for location based services in VANETs
Source: PLoS One. 2025 Jun 3;20(6):e0323438. doi: 10.1371/journal.pone.0323438 (PMC12132977; doi:10.1371/journal.pone.0323438)
Supplement: Attachment — Submitted filename: PONE-D-24-50794_Camerinelli.pdf [file pone.0323438.s001.pdf]

PLOS ONE

Blockchain Enabled Privacy Provisioning Scheme for Location Based Services in VANETs

--Manuscript Draft--

**Manuscript Number:**	PONE-D-24-50794
**Article Type:**	Research Article
**Full Title:**	Blockchain Enabled Privacy Provisioning Scheme for Location Based Services in VANETs
**Short Title:**	Blockchain Enabled Privacy Provisioning Scheme for Location Based Services in VANETs
**Corresponding Author:**	M. Usman Ashraf, Ph.D<br>GC Women University Sialkot<br>Sialkot, Punjab PAKISTAN
**Keywords:**	Blockchain; Location Base Service; VANET; Anonymization
**Abstract:**	In recent years, vehicular ad hoc networks (VANETs) have become a common fixture in intelligent traffic systems. The design of VANETs incorporates key features like autonomy, distributed networking, and a dynamic topology, all essential for their operation. These characteristics, along with VANET's role in enhancing road safety, have garnered significant interest from both industry and academia, particularly in endeavors aimed at improving transportation systems to potentially save lives. However, the secure broadcasting of messages in VANET, an open access system, remains a major and highly challenging issue regarding security and privacy. Numerous studies have proposed solutions for VANET security and privacy, yet few have comprehensively addressed overarching privacy requirements such as unobservability. To tackle these limitations, we introduce blockchain-based a novel privacy provisioning scheme to secure VANET communication system. Leveraging the privacy and efficiency attributes in anonymizer server, we use cache based anonymizer server configured between On-Board Unit (OBU) and Road side unit (RSU) that improve system privacy as well as efficiency. Through performance evaluation, our scheme not only satisfies the aforementioned security and privacy criteria but also proves resilient against various types of attacks, including replay, impersonation, modification, and man-in-the-middle attacks.
**Order of Authors:**	M. Usman Ashraf, Ph.D
	Iqra Ilyas
	Sami S. Albouq
	Khlood Shinan
	Hanan E. Alhazmi
	Fatmah Alanazi
	Saqib Ali
**Additional Information:**	
**Question**	**Response**
**Financial Disclosure**<br><br>Enter a financial disclosure statement that describes the sources of funding for the work included in this submission. Review the submission guidelines for detailed requirements. View published research articles from *PLOS ONE* for specific	The author(s) received no specific funding for this work.

Powered by Editorial Manager® and ProduXion Manager® from Aries Systems Corporation

examples.<br><br>This statement is required for submission and **will appear in the published article** if the submission is accepted. Please make sure it is accurate.<br><br>**Funded studies**<br>Enter a statement with the following details:<br>• Initials of the authors who received each award<br>• Grant numbers awarded to each author<br>• The full name of each funder<br>• URL of each funder website<br>• Did the sponsors or funders play any role in the study design, data collection and analysis, decision to publish, or preparation of the manuscript?<br><br>Did you receive funding for this work?	
**Competing Interests**<br><br>Use the instructions below to enter a competing interest statement for this submission. On behalf of all authors, disclose any competing interests that could be perceived to bias this work—acknowledging all financial support and any other relevant financial or non-financial competing interests.<br><br>This statement is required for submission and **will appear in the published article** if the submission is accepted. Please make sure it is accurate and that any funding sources listed in your Funding Information later in the submission form are also declared in your Financial Disclosure statement.<br><br>View published research articles from *PLOS ONE* for specific examples.	The authors have declared that no competing interests exist.

**NO authors have competing interests**<br><br>Enter: *The authors have declared that no competing interests exist.*<br><br>**Authors with competing interests**<br><br>Enter competing interest details beginning with this statement:<br><br>*I have read the journal's policy and the authors of this manuscript have the following competing interests: [insert competing interests here]*<br><br>* typeset	
**Ethics Statement**<br><br>Enter an ethics statement for this submission. This statement is required if the study involved:<br><br>• Human participants<br>• Human specimens or tissue<br>• Vertebrate animals or cephalopods<br>• Vertebrate embryos or tissues<br>• Field research<br><br>Write "N/A" if the submission does not require an ethics statement.<br><br>General guidance is provided below. Consult the submission guidelines for detailed instructions. **Make sure that all information entered here is included in the Methods section of the manuscript.**	N/A

**Format for specific study types**<br><br>**Human Subject Research (involving human participants and/or tissue)**<br>• Give the name of the institutional review board or ethics committee that approved the study<br>• Include the approval number and/or a statement indicating approval of this research<br>• Indicate the form of consent obtained (written/oral) or the reason that consent was not obtained (e.g. the data were analyzed anonymously)<br><br>**Animal Research (involving vertebrate animals, embryos or tissues)**<br>• Provide the name of the Institutional Animal Care and Use Committee (IACUC) or other relevant ethics board that reviewed the study protocol, and indicate whether they approved this research or granted a formal waiver of ethical approval<br>• Include an approval number if one was obtained<br>• If the study involved *non-human primates*, add *additional details* about animal welfare and steps taken to ameliorate suffering<br>• If anesthesia, euthanasia, or any kind of animal sacrifice is part of the study, include briefly which substances and/or methods were applied<br><br>**Field Research**<br><br>Include the following details if this study involves the collection of plant, animal, or other materials from a natural setting:<br>• Field permit number<br>• Name of the institution or relevant body that granted permission	
**Data Availability**<br><br>Authors are required to make all data underlying the findings described fully available, without restriction, and from the time of publication. PLOS allows rare exceptions to address legal and ethical concerns. See the PLOS Data Policy and FAQ for detailed information.	Yes - all data are fully available without restriction

A Data Availability Statement describing where the data can be found is required at submission. Your answers to this question constitute the Data Availability Statement and **will be published in the article**, if accepted.<br><br>**Important:** Stating 'data available on request from the author' is not sufficient. If your data are only available upon request, select 'No' for the first question and explain your exceptional situation in the text box.<br><br>Do the authors confirm that all data underlying the findings described in their manuscript are fully available without restriction?	
**Describe where the data may be found in full sentences. If you are copying our sample text, replace any instances of XXX with the appropriate details.**<br><br>• If the data are **held or will be held in a public repository**, include URLs, accession numbers or DOIs. If this information will only be available after acceptance, indicate this by ticking the box below. For example: *All XXX files are available from the XXX database (accession number(s) XXX, XXX.).*<br>• If the data are all contained **within the manuscript and/or Supporting Information files**, enter the following: *All relevant data are within the manuscript and its Supporting Information files.*<br>• If neither of these applies but you are able to provide **details of access elsewhere**, with or without limitations, please do so. For example:<br><br>*Data cannot be shared publicly because of [XXX]. Data are available from the XXX Institutional Data Access / Ethics Committee (contact via XXX) for researchers who meet the criteria for access to confidential data.*<br><br>*The data underlying the results presented in the study are available from (include the name of the third party*	All relevant data are within the manuscript and its Supporting Information files

and contact information or URL).<br>• This text is appropriate if the data are owned by a third party and authors do not have permission to share the data.<br><br>* typeset	
Additional data availability information:	

Manuscript Click here to access/download;Manuscript;paper.docx

Blockchain Enabled Privacy Provisioning Scheme for Location Based Services in VANETs

Iqra Ilyas[1], M. Usman Ashraf[2,*], Sami S. Albouq[3], Khlood Shinan[4], Hanan E. Alhazmi[5], Fatmah Alanazi[6], and Saqib Ali[7]

[1,2] Department of Computer Science, GC Women University Sialkot, Pakistan.
[3]Faculty of Computer and Information Systems, Islamic University of Madinah, Madinah, 42351, Saudi Arabia

[4]Department of Computers, College of Engineering and Computers in Al-Lith, Umm Al-Qura University, Makkah, Saudi Arabia
[5]Computer Science Department, College of Computer and Information Systems, Umm Al-Qura University, Makkah 21955, Saudi Arabia.
[6]Computer Science Department, College of Computer and Information Sciences, Imam Muhammad Bin Saud University, Riyadh 11432, Saudi Arabia.

[7]Department of Computer Science, University of Agriculture Faisalabad, Pakistan.

*Corresponding author: M. Usman Ashraf (e-mail: usman.ashraf@gcwus.edu.pk)

Abstract

In recent years, vehicular ad hoc networks (VANETs) have become a common fixture in intelligent traffic systems. The design of VANETs incorporates key features like autonomy, distributed networking, and a dynamic topology, all essential for their operation. These characteristics, along with VANET's role in enhancing road safety, have garnered significant interest from both industry and academia, particularly in endeavors aimed at improving transportation systems to potentially save lives. However, the secure broadcasting of messages in VANET, an open access system, remains a major and highly challenging issue regarding security and privacy. Numerous studies have proposed solutions for VANET security and privacy, yet few have comprehensively addressed overarching privacy requirements such as unobservability. To tackle these limitations, we introduce blockchain-based a novel privacy provisioning scheme to secure VANET communication system. Leveraging the privacy and efficiency attributes in anonymizer server, we use cache based anonymizer server configured between On-Board Unit (OBU) and Road side unit (RSU) that improve system privacy as well as efficiency. Through performance evaluation, our scheme not only satisfies the aforementioned security and privacy criteria but also proves resilient against various types of attacks, including replay, impersonation, modification, and man-in-the-middle attacks.

***Keywords**:* Blockchain; Location Base Service; VANET; Anonymization.

1. Introduction

Vehicle ad-hoc networks (VANETs) play a crucial role in Location-Based Services (LBS) systems, offering a myriad of significant advantages. The integration of VANETs with LBS systems revolutionizes the way we perceive and utilize location-based information. Firstly, VANETs provide real-time and accurate positioning data, leveraging the interconnectedness of vehicles and roadside infrastructure to constantly update and refine location information. This accuracy is paramount in applications such as navigation systems, emergency services, and traffic management, where precise location data is critical for decision-making. Moreover, VANETs enhance the efficiency of LBS systems by enabling dynamic routing and traffic optimization based on current traffic conditions, thereby reducing congestion and travel time. Additionally, VANETs facilitate the dissemination of location-specific information to vehicles in the vicinity, such as

nearby amenities, points of interest, and potential hazards, enhancing the overall driving experience and safety [1]. Furthermore, the seamless integration of VANETs with LBS systems opens up opportunities for innovative services, such as collaborative driving, autonomous vehicle coordination, and personalized location-based advertising. In essence, VANETs serve as the backbone of modern LBS systems, empowering them with unprecedented accuracy, efficiency, and functionality, ultimately revolutionizing the way we interact with our environment on the road. For instance, a vehicle equipped with VANET technology is utilizing a navigation system integrated with LBS capabilities. In this scenario, the VANET-enabled LBS system can provide real-time traffic updates and route suggestions based on current road conditions and user preferences [2]. However, the collection and processing of location data in VANETs raise concerns regarding user privacy disclosure. As vehicles transmit their location and trajectory information to the VANET infrastructure and other vehicles in the vicinity, there's a potential risk of unauthorized access or misuse of this data, leading to privacy breaches. To address this issue, VANET-enabled LBS systems must implement robust privacy protection mechanisms. These mechanisms could include encryption of location data during transmission, anonymization techniques to dissociate location information from specific vehicles, and access control mechanisms to restrict data usage to authorized entities only [3]. Moreover, transparent privacy policies should be communicated to users, detailing how their location data will be collected, processed, and shared within the VANET ecosystem. Users should have the option to control the level of granularity of location sharing and to opt-out of certain data collection practices if they so choose [4].

To overcome the privacy challenges in above scenario, blockchain technology holds significant promise in protecting user privacy within VANET Location-Based Service (LBS) systems [5-8]. By its nature, blockchain offers a decentralized and immutable ledger where transactions, in this case, location data exchanges, can be securely recorded and verified without the need for intermediaries. In a VANET LBS system, blockchain can ensure the integrity and confidentiality of location data by encrypting and storing it in tamper-proof blocks across the network. Therefore, we have proposed a novel Blockchain based privacy protecting approach for VANET system. Our contribution can be summarized as follows:

- To overcome the challenges in centralized systems, this study introduces blockchain technology and devises an innovative decentralized framework known as the blockchain-based VANET. Proposed approach not only guarantees the integrity and security of SBMs but also conserves storage space and reduces processing time within the blockchain.
- To address performance issues in existing distributed systems, we implement cache based anonymizer servers in our scheme to increase the efficiency of the system which is primary concern once a vehicle is connected to LBS system and waiting for query response. This method reduces the computational workload and minimizes the need for direct communication among vehicles, thus improving the privacy and security of vehicle data.
- We implement the third security layer known as CA which is responsible to authenticate the authorized accessibility of the system.
- We evaluate our scheme in open source OPNET and Veins vehicular network simulation framework which is a great platform to authenticate the proposed scheme.
- Finally, we evaluate the effectiveness of our scheme by quantifying different privacy metrics including communication cost, computation cost, probability of location leakage, cache hit ratio, and entropy.

Rest of the paper is organized in such way that section II describes the existing privacy provisioning state of the art approaches used in VANET systems. Further, section III presents the preliminaries and related technologies used in this research. Section IV demonstrates the problem definition and system architecture. In section V, we conduct the security analysis of proposed scheme, and present multiple lemmas against possible privacy attacks. Section VI explain the experimental setup, results and comprehensive discussion. Lastly, we conclude our study in section conclusion section and present all used references at the end of the paper.

2. Related Work

The issue of privacy arises when sensitive traffic-related messages are vulnerable to unauthorized access and require safeguarding against misuse or exposure. In the realm of vehicular communication, it is imperative to address privacy concerns across all stages of vehicle interaction, encompassing aggregation, processing, collection, evaluation, and visualization. Due to the sensitive nature of the information exchanged, preserving privacy is of utmost importance in this domain. Privacy preservation has garnered significant attention in recent VANET research endeavors. Scholars have delineated privacy protection into three primary categories: location privacy, trajectory privacy, and identity privacy. Extensive research has delved into this subject, and the most relevant studies are delineated below.

In terms of protective mechanisms, privacy preservation typically falls into two main types: anonymity-based [9] and encryption-based [10] approaches. Anonymity-based methods frequently employ k-anonymity mechanisms, originally devised to safeguard users' location privacy in LBS applications but now widely applied in VANET. By leveraging the principle of maximum entropy, researchers identify k appropriate vehicles with the closest historical request probability, concealing the real vehicle among them to protect vehicular privacy. On the other hand, encryption-based techniques are commonly utilized in VANET authentication, a crucial aspect ensuring the legitimacy of communications. Currently, VANET authentication predominantly adopts asymmetric encryption authentication methods such as Public Key Infrastructure (PKI) [11] and Elliptic Curve Digital Signature Algorithm (EDSA) [12], alongside symmetric encryption authentication mechanisms like group signature authentication [13].

For instance, one study [14] explored the distribution of vehicular pseudonyms utilizing fog computing technology, effectively managed by the edge resources of VANET to enhance location privacy. Similarly, another research effort [15] introduced a policy for trajectory privacy employing multiple mix zones, ensuring the unlinkability of pseudonyms and safeguarding vehicular trajectory privacy.

Moving on to the intersection of blockchain and VANET, blockchain technology has evolved through three generations, from its focus on digital currency transactions (Blockchain 1.0) to the registration, validation, and transfer of smart contracts (Blockchain 2.0), and finally transcending into broader applications encompassing various sectors (Blockchain 3.0). In recent years, researchers have introduced blockchain into VANET, capitalizing on its decentralized, redundant storage, collectively maintained, and tamper-proof characteristics. For instance, Joy. K, and Gerla. M. [16], proposed the concept of blocktree, where vehicles embed their signatures into the blockchain. Dorri, A., Kanhere, S. S., & Jurdak, R. [17], suggested a lightweight scalable blockchain for VANET, and based on this, proposed a blockchain architecture with decentralized privacy protection for intelligent vehicle systems. Moreover, Lei et al. [18] introduced a novel network topology based on blockchain structure to simplify distributed key management in heterogeneous vehicle communication systems. Additionally, blockchain has been proposed as a means for privacy-preserving proof of location in certain contexts [19].

Payattukalanirappel et al. [20] introduced a decentralized authenticated key agreement mechanism utilizing smart contracts to enhance the security of vehicular ad-hoc networks, constituting a VANETs authenticated key agreement scheme based on smart contracts. This scheme operates on a public Blockchain, eliminating the need for a Trusted Authority (TA) for key generation. Within this framework, Roadside Units (RSUs) form the Blockchain network, and smart contracts are executed on the Blockchain. The key agreement protocol incorporates the use of Bloom filters to verify whether public keys are generated by registered vehicles. However, the utilization of Bloom filters in this protocol introduces additional latency. The protocol involves the creation of four Blockchain transactions: one for vehicle registration, another for registering the vehicle's public key, a third for verifying the legality of the roadside unit, and the final transaction for registering the public key of the RSU. Despite these measures, the authentication and key agreement phase is susceptible to man-in-the-middle attacks, as any third party can intercept messages from the sender, fabricate them, and relay them to the receiver.

Similarly, Shen et al. [21] introduced a batch authentication protocol for the Internet of Vehicles, leveraging Blockchain technology. The system comprises several components including Trusted Authority, Road Side

Unit, Vehicle, Fog Server, and Cloud Server. The protocol encompasses vehicle-to-vehicle authentication as well as batch authentication, specifically vehicle-to-RSU authentication, where a group of vehicles is authenticated under a particular RSU. The Trusted Authority (TA) is responsible for delivering certificates containing identity, public key, and private key to all vehicles and RSUs. Upon receiving a partial block from the RSU, which includes a list of transactions and their compact signature, the fog node verifies the validity of the signature. If the signature is valid, the partial block is forwarded to the cloud server. Subsequently, the cloud server converts the partial block into a complete block and conducts mining on the block using the practical Byzantine Fault Tolerance (pBFT) consensus algorithm. However, the addition of the Cloud Server adds complexity to the system and consequently increases the overall latency of the system.

Tandon et al. [22] proposed a specialized vehicle-to-infrastructure handover authentication protocol designed for VANETs, leveraging Blockchain technology. The system incorporates various components, including the Trusted Authority (TA), Road Side Unit (RSU), vehicles, and Blockchain. In the initial registration phase, the TA deploys RSUs and issues smart cards to vehicles, with RSUs responsible for authenticating vehicles before any communication takes place. The Blockchain framework encompasses both the TA and RSUs. Following registration, the TA uploads data pertaining to the pseudo-identity of registered vehicles onto the Blockchain. Throughout the authentication process, RSUs verify the registration status of vehicles. Upon successful completion of vehicle-to-infrastructure authentication, RSUs initiate a transaction on the Blockchain, comprising the vehicle's temporal identity, a randomly generated number, and the RSU's signature. It is worth noting that while the paper delves into vehicle-to-infrastructure communication, it fails to address the critical aspect of V2V communication within the VANET framework.

The literature analysis outlined above highlights the prevalent challenges faced by existing systems, notably concerning substantial computational and communication burdens. Achieving an optimal equilibrium between communication and computation costs remains a rarity. Moreover, some systems depend on multiple trusted third parties, demanding trust from both users and infrastructure, resulting in heightened communication overhead and latency. Additionally, reliance on centralized databases in certain systems introduces transparency deficiencies and single points of failure risks. The proposed protocol seeks to address and alleviate these identified issues.

3. Preliminaries

A. Blockchain technologies

Blockchain technology stands as a pivotal emerging technology with far-reaching implications across various sectors. Its significance lies in its ability to revolutionize traditional systems by providing decentralized, transparent, and immutable records of transactions. One of its primary applications is in finance, where it enables secure and efficient peer-to-peer transactions without the need for intermediaries. Beyond finance, blockchain finds utility in supply chain management, ensuring transparency and traceability of goods from production to consumption. Additionally, its decentralized nature holds promise for enhancing data security and privacy in healthcare, voting systems, and identity management. Moreover, blockchain facilitates the creation of decentralized applications (DApps) and smart contracts, automating processes and reducing reliance on centralized authorities. As blockchain technology continues to evolve, its potential to disrupt existing paradigms and foster innovation across diverse industries solidifies its status as a transformative force in the digital era. Blockchain technology, denoted by *B*, is increasingly utilized for privacy protection in various domains due to its inherent characteristics such as decentralization, immutability, and transparency. In a blockchain-based privacy protection scheme, each participant, denoted by *Pi*, possesses a unique cryptographic key pair (*SKi*,*PKi*) where *SKi* represents the private key and *PKi* denotes the public key. Transactions, denoted by *T*, are securely recorded on the blockchain using cryptographic hashing functions, ensuring data integrity and preventing tampering. Privacy protection is achieved through the use of pseudonyms, denoted by Pseudo*i*, which are derived from the public key *PKi* and used to conceal the true identity of participants. Smart contracts, denoted by *SC*, facilitate the enforcement of privacy policies and consent management, dictating how data is accessed and utilized. Moreover, zero-knowledge proofs, denoted by *ZKP*, enable participants to prove the validity of a statement without revealing any sensitive information.

Through the integration of these cryptographic techniques and decentralized consensus mechanisms such as Proof of Work (PoW) or Proof of Stake (PoS), blockchain technology ensures robust privacy protection while preserving data transparency and integrity in a trustless environment. The basic architecture of blockchain is presented in figure 1 as follows.

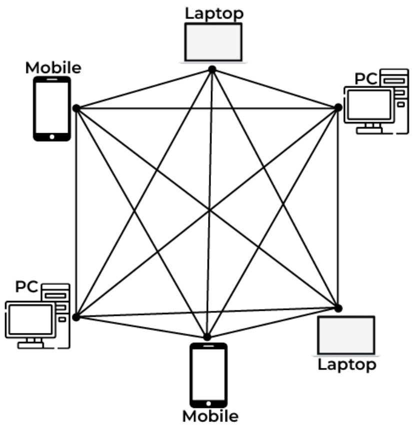

Figure 1. Fundamental architecture of blockchain.

B. K-Anonymity Approach

K-anonymity is a fundamental concept in privacy-preserving data anonymization, aiming to mitigate the risk of individual re-identification in datasets containing sensitive information. It provides a robust framework for privacy protection in datasets containing sensitive information. By ensuring that each individual's data is sufficiently anonymized and grouped with similar records, k-anonymity helps to protect against privacy breaches and unauthorized disclosure of personal information. At its core, k-anonymity ensures that each data record remains indistinguishable from at least $k-1$ other records with respect to certain attributes. This means that for any given data record di in a dataset D, there exists at least one other record dj sharing the same sensitive attributes S as di, where the total number of such records $|dj|$ is equal to or greater than k. Mathematically, this concept is expressed in equation 1 as follows:

$$\forall di \in D, \exists dj \in D \text{ s.t. } di\,[S] = dj[S] \qquad (1)$$

where $|dj| \geq k$

To put it simply, k-anonymity ensures that individual records in a dataset are grouped together with enough similar records to obscure the identity of any specific individual. This makes it significantly more challenging for adversaries to pinpoint and re-identify individuals based on sensitive attributes alone. Achieving k-anonymity involves applying various anonymization techniques to the dataset. Generalization involves replacing specific attribute values with more generalized ones, such as replacing exact ages with age ranges. Suppression, on the other hand, entails removing certain attributes entirely from the dataset to prevent identification. Perturbation techniques add noise or randomization to the data, making it more difficult for adversaries to infer specific details about individuals.

C. Dynamic threshold encryption (DTE)

Dynamic threshold encryption (DTE) is a cryptographic scheme that allows for the secure sharing of a secret among a group of participants, with the ability to dynamically adjust the threshold for decryption. It provides a flexible and adaptable approach to secure data sharing among multiple participants by allowing the threshold for decryption to be dynamically changed as needed. In DTE, a secret S is encrypted using a public key derived from shares distributed among n participants. Unlike traditional threshold encryption schemes where a fixed number of shares are required for decryption, DTE enables the threshold value t to be dynamically changed based on certain conditions or criteria. DTE consist of four primary steps discussed as follows:

1. ***Key Generation:***
   - Each participant i generates a pair of public-private keys (PKi,SKi).
   - Each participant i calculates their share $Share_i$ using their private key SK_i.
2. ***Encryption:***
   - The secret S is encrypted using a public key PK derived from the shares: $E(S)=Encrypt(S,PK)$
3. ***Decryption:***
   - To decrypt the encrypted secret E(S), at least t participants need to collaborate.
   - Suppose T is the set of indices of the participants who collaborate for decryption, where $|T|\geq t$.
   - The combined private keys of the collaborating participants are used to recover the secret: $S=Decrypt(E(S),\{SKi|i\in T\})$
4. ***Dynamic Threshold Adjustment:***
   - The threshold value t can be dynamically adjusted based on specific conditions or events.
   - For example, if the number of active participant's changes, the threshold value can be recalculated to ensure the required level of security.

4. Problem Definition and System Architecture

This section commences with an overview of our system structure followed by the problem definition. Next, we introduce the extended blockchain tailored for VANETs. Subsequently, we describe a succinct overview of registration process along with authentication procedure employed in the proposed trust management model. Finally, we delineate several fundamental assumptions crucial for ensuring the seamless operation of the system.

A. Problem Definition

In VANET systems, ensuring privacy for vehicular communication is of paramount importance to prevent unauthorized access to sensitive information and protect user anonymity. Let $G=(V,E)$ denote a VANET network, where V represents the set of vehicles and E represents the set of communication links between vehicles. Each vehicle vi in V is associated with a set of attributes denoted by $Attr(vi)$, including location, speed, direction, and identity information. The goal is to design privacy provisioning mechanisms that preserve the privacy of vehicle attributes while allowing for efficient and secure communication within the network. Further it can be formulated as follows:

Attribute Confidentiality: Ensure that sensitive attributes of vehicles, such as location and identity, are kept confidential and cannot be intercepted or inferred by unauthorized entities. $\forall vi,vj\in V$, if $Attr(vi)=Attr(vj)$, then vi and vj should be indistinguishable to external observers.

Communication Security: Guarantee secure communication between vehicles to prevent eavesdropping, tampering, and message interception by malicious entities. $\forall eij \in E$, ensure that messages exchanged between vi and vj are encrypted and authenticated to maintain communication security.

Anonymity Preservation: Preserve the anonymity of vehicles in the network to prevent the tracking and profiling of individual vehicles by adversaries. $\forall vi\in V$, ensure that the identity of vi remains hidden from external observers to maintain anonymity.

Efficiency and Scalability: Design efficient and scalable privacy provisioning mechanisms that can be implemented in large-scale VANET networks with minimal computational and communication overhead. Minimize computational and communication costs associated with privacy provisioning while ensuring scal ability to accommodate large numbers of vehicles in the network. Minimize computational and communication costs associated with privacy provisioning while ensuring scalability to accommodate large numbers of vehicles in the network.

B. System Architecture

To protect vehicle's identity and spatial information, we propose a blockchain based VANET system as shown in figure 2. The system illustrates the network model that contain different components including

vehicles, OBUs, anonymizer server, RSUs, core network (that further include CA, LBSP, CSP), blockchain (BC), and blockchain network (BCN).

Mobile Entities (Vehicles)

In a Vehicular Ad-Hoc Network (VANET) system, vehicles play a pivotal role as mobile nodes that dynamically form a communication network. Vehicles are represented as a set $V=\{v1,v2,...,vn\}$, where n denotes the total number of vehicles. Each vehicle vi is equipped with an On-Board Unit (OBU), denoted as $OBUi$, responsible for facilitating communication within the network. Vehicles play a crucial role in exchanging information, such as Safety Beacon Messages (SBMs), with other vehicles and Road Side Units (RSUs) to enhance safety and efficiency on the road. The communication capabilities of vehicles are essential for enabling various VANET applications, including collision avoidance, traffic management, and emergency assistance. Mathematically, the presence of vehicles in the VANET system can be expressed as $V=\{v1,v2,...,vn\}$, where each vehicle is equipped with $OBUi$ to participate in communication protocols and data exchange.

On Board Unit

In the VANET system, the On-Board Unit (OBU) serves as a critical component for each vehicle vi. Mathematically, we represent the set of OBUs as $O=\{OBU1,OBU2,...,OBUn\}$, where n denotes the total number of vehicles in the network. The OBU $OBUi$ associated with each vehicle facilitates communication and data exchange within the VANET. It enables vehicles to transmit and receive Safety Beacon Messages (SBMs) and other relevant information to enhance road safety and efficiency. The OBU is responsible for implementing communication protocols, such as Vehicle-to-Vehicle (V2V) and Vehicle-to-Infrastructure (V2I) communication, and ensuring secure and reliable transmission of data. Mathematically, the presence of OBUs in the VANET system can be expressed as $O=\{OBU1,OBU2,...,OBUn\}$, where each OBU $OBUi$ is essential for enabling vehicle communication and supporting VANET functionalities.

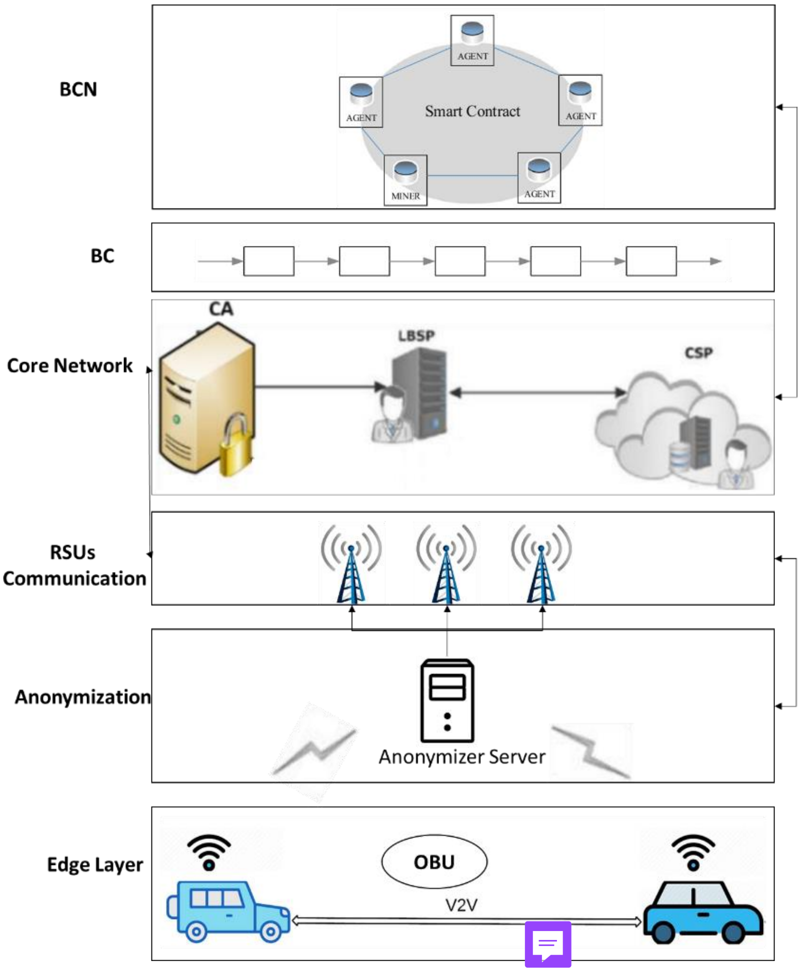

Figure 2. Block diagram of proposed privacy provisioning for VANETs

Algorithm 1: On-Board Unit Operations

Inputs:
query: Query or message from the vehicle
cachedData: Cached data stored in the OBU
Output:
Qresponse: Response to the query or message
Procedure:
1. Ensure that the query is not empty: require bytes(query).length>0
2. Check if the query can be answered using cached data: if query is present in cachedData then
3. Retrieve the response from the cached data: response=getCachedResponse(query)
4. Return the response: return response
5. If the query is not found in the cached data: else
6. Forward the query to the Anonymizer Server for further processing: response=sendToAnonymizer(query)
7. Cache the response for future queries: cacheResponse(query,response)
8. **Return** the response: return Qresponse

Anonymizer Server

In term of privacy provisioning, the Anonymizer Server plays a vital role in preserving privacy by anonymizing the identity and location information of vehicles. Mathematically, we denote the Anonymizer Server as A. It is linked with each On-Board Unit (OBU) $OBUi$ associated with vehicles in the network. The link between the Anonymizer Server and the OBU is represented as a function $O \rightarrow A$, where O is the set of OBUs and A is the Anonymizer Server. The Anonymizer Server receives sensitive information, such as location data and vehicle identifiers, from the OBUs. It then applies anonymization techniques to obfuscate this information before forwarding it to other entities in the network. By anonymizing data at the source, the Anonymizer Server helps protect the privacy of vehicles and their occupants while enabling efficient communication within the VANET system. Mathematically, the link between the OBUs and the Anonymizer Server can be expressed as $f{:}O \rightarrow A$ indicating the association between each OBU and the Anonymizer Server for privacy protection purposes.

Road Side Units (RSUs)

Road Side Units (RSUs) play a crucial role in facilitating communication and providing infrastructure support for vehicles. Mathematically, we denote the set of RSUs as $R=\{RSU1,RSU2,...,RSUm\}$, where m represents the total number of RSUs deployed in the network.

Algorithm 2: Anonymization Operations

Inputs:
vehicleData: Vehicle data to be anonymized
cloakingRegion: Cloaking region information

Output:
anonymizedData: Anonymized data

Procedure:
1. Ensure that the vehicle data is not empty: require bytes(vehicleData).length>0
2. Ensure that the cloaking region information is provided: require cloakingRegion≠null
3. Check the validity of the vehicle data against the cloaking region: if isValid(*vehicleData*,cloakingRegion) then

4. Anonymize the vehicle data using the specified cloaking region: anonymizedData=anonymize (*vehicleData*, cloakingRegion)
5. **Return** the anonymized data: return anonymizedData

Each RSU *RSUi* is strategically positioned along roadsides to cover specific geographical areas. RSUs serve as communication hubs, enabling Vehicle-to-Infrastructure (V2I) communication and enhancing connectivity within the VANET. They relay information between vehicles and the infrastructure, such as traffic updates, road conditions, and safety alerts. Mathematically, the presence of RSUs in the VANET system can be expressed as $R=\{RSU1,RSU2,...,RSUm\}$, where each RSU *RSUi* contributes to the network's functionality by providing infrastructure support and enabling communication between vehicles and the roadside infrastructure. Algorithm 3 presents the registration process of RSUs.

Algorithm 3: Registration process of RSUs in VANET

Inputs:
rsuID: ID of the RSU
Output:
bool: Boolean indicating the success of the registration
Procedure:
1. Ensure that the sender's address is not null: $msg_{sender} \neq$ address(0)
2. Ensure that the RSU's address in the mapping is not the sender's address require $RSU_{mapping}$ [msg_{sender}]. rsuAddress$\neq msg_{sender}$
3. Ensure that the length of the RSU ID is greater than zero: bytes(rsuID).length>0
4. Ensure that the sender's vehicle is not already registered: $vehicle_{Mapping}$[msg_{sender}]. isRegd =false
5. Set the RSU's address in the mapping to the sender's address: $RSU_{mapping}$(msg_{sender}).rsuAddress=msg_{sender}
6. Set the RSU's ID in the mapping: $RSU_{Mapping}$[msg_{sender}].id= rsuID
7. Mark the RSU as registered: rsuMapping[msg_{sender}].isRegd=true
8. **Return** the boolean indicating successful registration: return bool

Core Network Layer

In the VANET system, the core network layer comprising the Certificate Authority (CA), Location-Based Service Provider (LBSP), and Cloud Service Provider (CSP) plays a major role in managing and processing data received from Road Side Units (RSUs) before forwarding it to the blockchain layer. We denote the components of the core network layer as $C=\{CA,LBSP,CSP\}$. Upon receiving data from RSUs, the CA, as a trusted entity, verifies the authenticity of the data and issues digital certificates to ensure secure communication within the network. The LBSP, responsible for location-based services, processes location data received from RSUs to provide relevant services to vehicles, such as navigation assistance and traffic updates. The CSP, serving as a cloud-based platform, stores and manages data received from RSUs, ensuring scalability and accessibility. Mathematically, the functions of the core network layer can be expressed as $C=\{CA,LBSP,CSP\}$, where each component contributes to data processing, verification, and management before forwarding it to the blockchain layer for further processing and storage.

Blockchain (BC), and Blockchain Network (BCN)

In the VANET system, Blockchain (BC) serves as a decentralized and immutable ledger to protect the privacy of vehicles by securely storing and managing sensitive information. Mathematically, we denote the Blockchain as *BC*, which is further linked with the Blockchain network (BCN). The Blockchain network consists of a distributed network of nodes responsible for validating and storing transactions. The role of BC in VANET is crucial for ensuring the integrity and privacy of data exchanged between vehicles and infrastructure. BC utilizes cryptographic techniques, such as hashing and digital signatures, to secure transactions and prevent unauthorized access. By linking with the BCN, BC ensures transparency, decentralization, and tamper-resistance, making it an ideal platform for preserving the privacy of vehicle data. Mathematically, the association between BC and BCN can be represented as $BC \rightarrow BCN$, indicating

the linkage between the Blockchain and its network for protecting the privacy of vehicle information in the VANET system.

C. Workflow of Proposed model

Addressing privacy concerns within the blockchain-based VANET system necessitates meticulous control over all participating nodes. Hence, this study advocates the implementation of multiple privacy protection mechanisms at different stages to ensure the privacy provisioning to the user's sensitive information. For instance, anonymizer server (AS) are deployed at initial stage to anonymize the vehicle's location/identity information once it is forwarded to RSU through OBU. RSU point could be harmful to disclose the sensitive information. Anonymizer server generate the pseudonym data of received information from OBU and forwarded to RSU unit along with cloaking region. In this way, a secured path is initiated at first stage. In next stage, the the anonymized information is disseminated over the core network where CA generate a certificate against the received query and forward to LBSP module. Further, our proposed model advocates for the implementation of a private chain to construct the blockchain network. Within this framework, the hash values of all data within the core network are securely stored within the blockchain network. This measure serves as protection against tampering and manipulation by malicious entities. Any alteration to the data results in a change in its hash value, readily detectable within the auditable blockchain. Moreover, by storing only the hash values, considerable storage resources within the blockchain network are conserved, consequently enhancing system responsiveness. Smart contracts constitute another integral aspect of the proposed solution. These contracts embody predefined rules and conditions that are automatically executed once triggered. In this context, specific rules pertaining to authentication, k-anonymity, sub-identity generation and dynamics, SBMs recording, and more are encoded into smart contracts. By functioning solely based on code, smart contracts not only reduce transaction costs but also enhance accuracy and efficiency by obviating the need for human intervention. Agent nodes play a pivotal role within the blockchain network as participating entities responsible for maintaining the integrity of transactions through consensus mechanisms. Each agent node contributes to the consensus process and maintains a backup of the blockchain network's data. In this study, agent nodes adopt a Proof of Work (PoW) approach for achieving consensus. Furthermore, within the realm of agent nodes, a subset known as miners holds a distinct position. Miners are agent nodes that successfully solve mathematical problems and thus earn the privilege of maintaining legal blocks within the blockchain. These miners are tasked with processing new blocks and validating data before updating the blockchain network's backup data. As such, miners not only participate in the consensus process but also possess the capability to mine and validate new blocks, rendering them instrumental in the network's operation.

5. Security Analysis

This section illustrates that the proposed scheme effectively fulfills the security and privacy criteria outlined for vehicular communication in the design objectives subsection. Following are possible attacks and their resistance using our proposed scheme.

A. Query content modification attack resistance

Let S represent the set of legal vehicle-related sensitive information, and let $'S'$ represent the set of pseudonym data forwarded by the anonymizer server. Additionally, let C represent the cloaking region generated by the anonymizer server and forwarded to the RSU. Then, the lemma can be expressed as follows:

Lemma: For any adversary A attempting to tamper with the legal vehicle-related sensitive information S, such that $S \neq S'$, in the presence of the anonymizer server and the proposed scheme, the adversary's ability to modify S is significantly impeded. Mathematically, this can be represented as:

$$\forall S \in S, \forall A : P\,(A \text{ modifies } S \mid S \neq S', C) \ll P\,(A \text{ modifies } S \mid S = S', C) \qquad (2)$$

Where $P\,(A \text{ modifies } S \mid S \neq S', C)$ denotes the probability of the adversary A successfully modifying the legal vehicle-related sensitive information S when S is different from the pseudonym data $'S'$ and the cloaking region C is present. $P\,(A \text{ modifies } S \mid S = S', C)$ denotes the probability of the adversary A

successfully modifying the legal vehicle-related sensitive information *S* when *S* matches the pseudonym data *′S′* and the cloaking region *C* is present.

B. Content Replay attack resistance

For any attacker *A* attempting to perform a replay attack by using a different timestamp *′T′* in place of the original timestamp *T*, the proposed system effectively detects and rejects the replayed message. This can be represented as:

$$\forall T, T' \in \text{Timestamps}, \forall A: P\ (A\ T') \ll P\ (A\ \ T) \qquad (3)$$

Where *P* (*A* successfully replays message with *T′*) denotes the probability of the attacker *A* successfully replaying a message with a different timestamp *′T′*. *P* (*A* successfully replays message with *T*) denotes the probability of the attacker *A* successfully replaying a message with the original timestamp *T*. This proof indicates that the proposed system effectively mitigates replay attacks by verifying the freshness of timestamps in traffic-related messages. Since using a different timestamp results in a different value for the inequality check, replayed messages are promptly detected and rejected.

C. Impersonation attack resistance

Let *CA* represent the Certificate Authority in the core network of the VANET system. The lemma against impersonation attack resistance can be formulated as follows:

Lemma: The proposed VANET system, utilizing a Certificate Authority (CA) for query authentication, effectively resists impersonation attacks. Mathematically, this can be expressed as:

$$\forall \text{attacker } A, P\ (A) \ll P\ (\text{CA}) \qquad (4)$$

Where, *P*(*A* successfully impersonates as a legitimate user) denotes the probability of an attacker *A* successfully impersonating as a legitimate user without being detected. *P*(legitimate user successfully authenticates with CA) denotes the probability of a legitimate user successfully authenticating with the Certificate Authority (CA) to obtain valid credentials. This lemma asserts that the use of the Certificate Authority (CA) significantly reduces the likelihood of impersonation attacks in the VANET system. Legitimate users' authentication through the CA adds an extra layer of security, making it challenging for attackers to successfully impersonate legitimate users without valid credentials.

D. Middle-man attack resistance

Let *S* denote the sender of a message, *V* denote the verifier, and *M* represent the message transmitted in the VANET system. The lemma for Resistance to Man-in-the-Middle (MITM) Attack in the described scenario can be formulated as follows:

Lemma: In the VANET system architecture described, incorporating an anonymizer server connected with the On-Board Unit (OBU), Certificate Authority (CA) within the core network containing the Location Based Service Provider (LSP), and cloud service provider (CSP), followed by linkage with blockchain and further blockchain network, the system effectively resists Man-in-the-Middle (MITM) attacks. Mathematically, this can be expressed as:

$$\forall M, S, V : P\ (SV \text{ relationship is verified}) = 1 \qquad (5)$$

Where *P* (*SV* relationship is verified) denotes the probability that the relationship between the sender *S* and the verifier *V* is verified, ensuring that the message *M* is transmitted securely without intervention. *M* represents the message transmitted in the VANET system. It shows that in the described system architecture, the verification of the relationship between the sender and the verifier is a necessary condition for ensuring message validity and authenticity. By establishing this relationship and ensuring that a genuine message cannot be changed or fabricated, the system effectively mitigates the risk of Man-in-the-Middle (MITM) attacks.

6. System Evaluation, Results and Discussion

This section presents the effectiveness of our proposed blockchain based privacy provisioning model with respect to vehicle privacy and unity. The initial segment delineates the simulation environment, while the subsequent portion scrutinizes simulation outcomes utilizing four key metrics: computation cost, communication cost, probability of privacy leakage, and entropy. To ascertain the efficacy of our proposed architecture, we juxtapose it with well-known existing state-of-the-art methods including centralized system [12] and decentralized framework [21], and cache based centralized [22]. The primary disparity between the distributed architecture and our proposed framework lies in the data privacy layers, and data handling. In the blockchain network of the distributed architecture, SBMs data is directly stored, whereas in the blockchain network of our proposed architecture, we store the hash values of the SBMs data. Figure 3 describes the deployment of blockchain based smart contract in proposed privacy provisioning method.

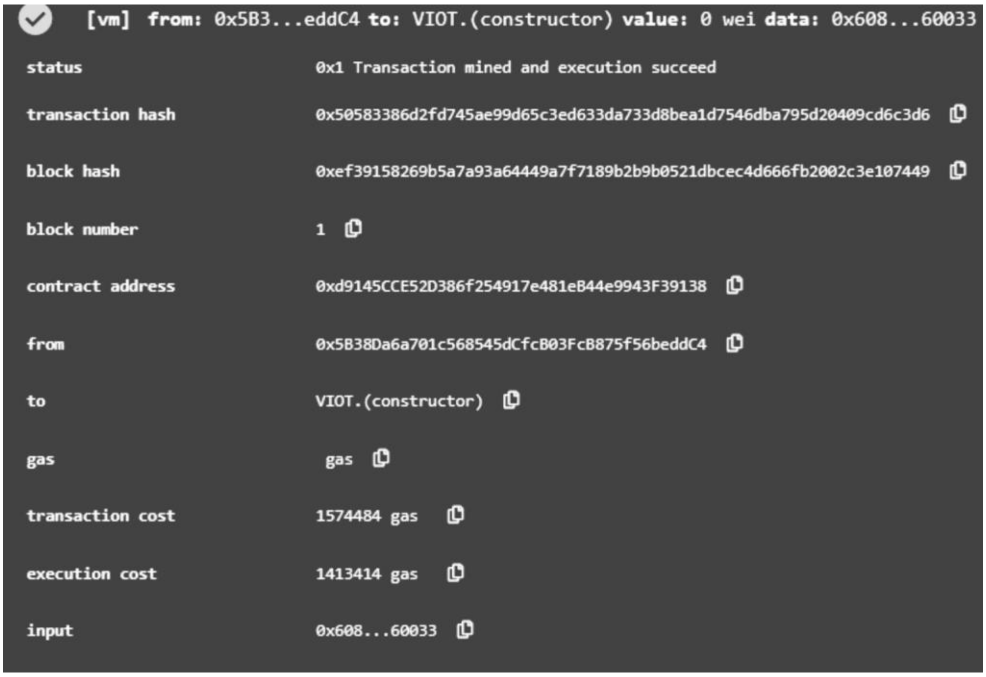

Figure 3: The deployment of blockchain based smart contract in proposed privacy provisioning method.

To optimize the simulation experiment, we segment our proposed architecture into three primary parts: (1) a cache-based anonymizer server, (2) the VANET network, and (3) the blockchain network. The cache-based anonymizer server serves as the initial layer, interfacing with OBUs to protect the privacy-related information by anonymizing actual data. Additionally, it efficiently handles query responses from the server cache, promptly delivering results when the desired information is accessible. This approach significantly reduces the computational and communication overhead of the entire system. The VANET network primarily involves vehicles uploading SBMs, while the blockchain network is responsible for recording transactions. We utilize OPNET [23] and the Veins framework [24] for simulating the VANET and blockchain networks, respectively. The Veins framework, being an advanced open-source vehicular network simulation framework, facilitates the implementation of blockchain-based computing platforms, incorporating smart contracts and the PoW consensus mechanism. In our experiments, we leverage the Veins platform to establish rules for authentication, k-anonymity unity, sub-identity dynamic change rules, and SBMs recording within smart contracts. For the blockchain network, we employ the PoW consensus to validate new data blocks. During experimentation, we simulate a target vehicle U navigating a trajectory Tr, comprising 10 locations {l1, l2,…,l10}. To ensure privacy, a corresponding undirected graph is generated for each location, resulting in 10 undirected graphs {G1, G2,…, G5} for vehicle U. We conduct experiments eight times to accurately assess performance, calculating average values for system time, average distance, connectivity, and privacy leakage entropy. Additionally, we simulate two traffic

scenarios, light and heavy, with the heavy traffic scenario involving a traffic density of 0.5 v/h (i.e., 5000 vehicles passing through Tr in one hour), while the light traffic scenario involves a density of 0.1 v/h.

A. Computation Cost

To evaluate our proposed privacy provisioning framework, we evaluate different metrics where computation cost is the first one that refers to the computational resources required to perform various operations such as encryption, decryption, hashing, verification, and other processing tasks. This cost is crucial to consider as it directly impacts the efficiency and performance of the system. Mathematically, the computation cost *C* can be expressed as the sum of the costs associated with individual operations:

$$C(comp) = \sum_{i=1}^{n} C_i \qquad (6)$$

Where, *C(comp)* is the total computation cost, *n* is the total number of operations, and C_i represents the cost of each individual operation. The computation cost for different operations can vary depending on factors such as algorithm complexity, key length, message size, and processing power of the devices involved. To calculate the computation cost in VANET systems, one must consider the specific operations involved in tasks such as encryption for securing communications, hashing for data integrity verification, signature generation and verification for authentication, and any other cryptographic operations necessary for privacy protection and security. Additionally, factors such as the number of vehicles, the frequency of message exchanges, and the computational capabilities of the network nodes need to be taken into account for accurate computation cost estimation. The computation cost analysis is illustrated in Figure 4 as outlined below. In terms of V2V communication, our scheme demonstrates a significantly lower computational cost compared to the referenced schemes, with a value of 0.23972. This indicates a substantial reduction in computational overhead, enhancing efficiency. Similarly, for V2RSU communication, our scheme exhibits a substantially lower computational cost of 0.13166 compared to the referenced schemes, further emphasizing the efficiency gains achieved. These results suggest that our proposed scheme effectively minimizes computational costs, making it a promising solution for VANET systems.

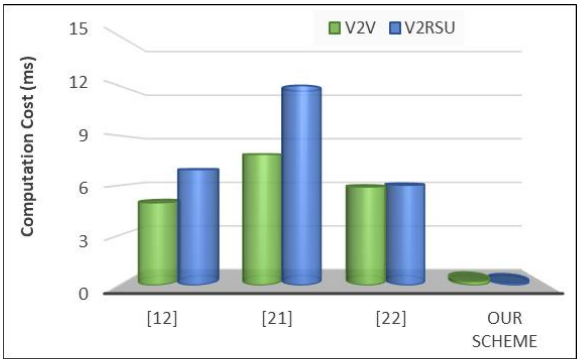

Fig: 4 Computation cost for V2V and V2RSU

B. Communication Cost

In our experiments, the second evaluation metric is communication cost that refers to the resources expended in transmitting data between vehicles, roadside units (RSUs), and other network components. It encompasses factors such as bandwidth usage, message overhead, and transmission delays. Mathematically, the communication cost *C(com)* is expressed in equation 7 as follows:

$$C(com) = \sum_{i=1}^{n} (B_i \text{ X } T_i) \qquad (7)$$

Where, *Bi* represents the bandwidth consumption of the ith communication link, *Ti* denotes the transmission time for data over the ith link, and *n* is the total number of communication links in the network.

In our experiments, analysis of communication cost evaluation across various schemes, including references [12], [21], and [22], compared to our proposed scheme, reveals significant differences in resource utilization. According to figure 5, in the case of V2V communication, our scheme demonstrates notably lower communication costs, with 534 and 530 bits compared to 1664, 2912, and 2560 bits for references [12], [21], and [22], respectively. Similarly, for V2RSU communication, our scheme exhibits reduced communication costs, registering values of 530 compared to 1920, 4416, and 2880 in references [12], [21], and [22], respectively. These findings underscore the efficiency and optimization achieved in communication resource utilization through our proposed scheme, suggesting its superiority in minimizing communication overhead in VANET systems.

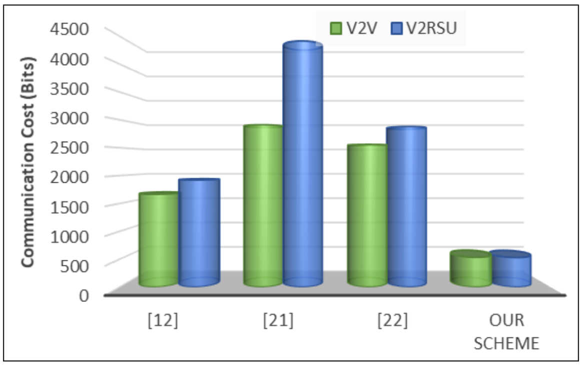

Fig 5: communication cost

C. Cache Hit Ratio

We evaluate the effective usage of cache while receiving queries from the vehicles. In a VANET system employing a cache-based anonymizer server situated between OBUs and RSUs, the cache hit ratio R_{hit} represents the proportion of queries successfully resolved from the cache compared to the total number of queries made. It can be expressed as:

$$R_{hit} = \frac{N_{hit}}{N_{query}} \quad (8)$$

Where, N_{hit} is the number of queries successfully resolved from the cache, and N_{query} is the total number of queries made by OBUs to the anonymizer server. Figure 6 presents the impact of cache usage that significantly improve the overall system. We observe that at initial level, cache hit ratio is almost in all methods, but a significant difference once the number of queries increased.

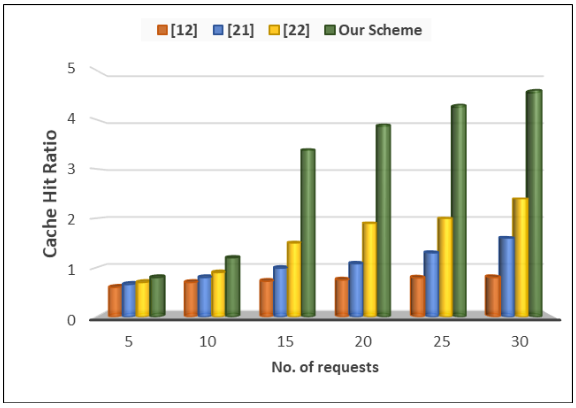

Fig 6: Cache hit ratio

D. Location Leakage Probability (L2P)

To calculate the location leakage probability in VANET systems, we define it as the probability that an adversary can accurately infer the location of a vehicle based on the information available to them. Mathematically, it can express as:

$$P\ (Loc\ Leakage\ = \frac{No.of\ successful\ loc\ interference}{Total\ no.of\ requests} \qquad (9)$$

Where *P* (Location Leakage) is the location leakage probability, "Number of Successful Location Inferences" represents the instances where the adversary correctly identifies the location of the vehicle, and "Total Number of Location Queries" denotes the total number of times the adversary attempts to infer the location of the vehicle. This evaluation can be performed over a given period or set of scenarios to assess the effectiveness of privacy protection mechanisms in the VANET system. As depicted in Figure 7, the probability L2P stands at 0.15 with centralized architecture, ranging between 0.1 and 0.12 with distributed architecture, and between 0.1 and 0.13 with cache-based centralized architecture. In contrast, our proposed architecture lowers the probability to approximately 0.05%. Consequently, the effectiveness of L2P protection is significantly enhanced with our proposed architecture.

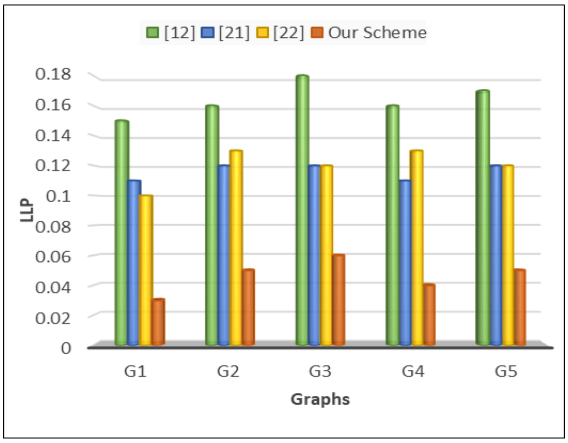

Fig 7: Location leakage probability

E. Entropy

In the experiments, we evalute our last metric entropy denoted as *H*(*X*) that represents the measure of uncertainty or randomness in a system. In the context of VANET systems, entropy is often used to quantify the level of privacy risk associated with the dissemination of data. The calculation of entropy involves the probability distribution of different states or outcomes within the system. Mathematically, entropy is calculated using the formula:

$$H(X) = -\sum_{i=1}^{n} p(x_i).log_2(p(x_i)) \qquad (10)$$

Where, *H*(*X*) is the entropy of the system *X*. $p(x_i)$ represents the probability of the ith state or outcome occurring. *n* is the total number of possible states or outcomes in the system.

By analyzing the frequency or likelihood of different information states, such as vehicle locations or identities, entropy provides insights into the level of privacy protection or risk within the system. Higher entropy values indicate greater uncertainty or randomness, while lower values suggest more predictable or deterministic behavior, which may pose privacy concerns in VANET environments. Figure 8 shows that our proposed scheme outperformed the existing methods and achieve maximum entropy level up to 5.

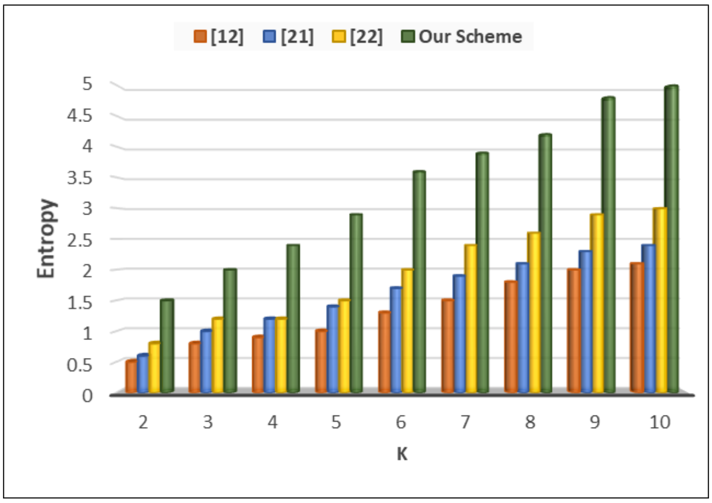

Fig 8: Anonymous entropy in k-anonymity mechanism

7. Conclusion, and Future Directions

The proposed blockchain based privacy provisioning scheme for VANET systems introduces a multi-layered approach aimed at protecting sensitive information against potential threats. By incorporating various privacy protection mechanisms at different stages, the system ensures robust privacy provisioning throughout. Initially, anonymizer servers (AS) anonymize vehicle location and identity information, enhancing security during data transmission to RSUs via OBUs. This anonymization process, coupled with the incorporation of cloaking regions, establishes a secure pathway at the outset. Subsequently, within the core network, Certificate Authorities (CAs) generate certificates to authenticate queries received, adding another layer of protection. Moreover, the deployment of a private chain within the blockchain network ensures secure storage of hash values, thwarting tampering attempts effectively. Smart contracts further fortify the system by automating predefined rules and conditions, minimizing human intervention while enhancing accuracy and efficiency. Agent nodes, particularly miners, play a vital role in maintaining the integrity of transactions through consensus mechanisms, contributing to the system's robustness. The proposed solution demonstrates resilience against various attacks, including query content modification, content replay, impersonation, and middle-man attacks, as evidenced by mathematical formulations and proofs. Through simulation experiments, the effectiveness of the proposed architecture is evaluated against centralized and decentralized approaches, highlighting significant improvements in computation cost, communication efficiency, cache hit ratio, location leakage probability, and entropy. The results demonstrate the superiority of the proposed scheme in minimizing computational overhead, optimizing resource utilization, enhancing privacy protection, and maximizing entropy levels, making it a promising solution for ensuring privacy in VANET systems.

Though in this research, our scheme outperforms the existing state of the art studies, however, by future perspectives, our aim is to implement the proposed scheme in real environment to know the effectives in real scenarios.

Conflicts of Interest

The authors declare that they have no conflicts of interest.

8. References

[1]. Yadav, V. K., Verma, S., & Venkatesan, S. (2020). Efficient and secure location-based services scheme in VANET. IEEE Transactions on Vehicular Technology, 69(11), 13567-13578.
[2]. Cui, J., Wen, J., Han, S., & Zhong, H. (2018). Efficient privacy-preserving scheme for real-time location data in vehicular ad-hoc network. IEEE Internet of Things Journal, 5(5), 3491-3498.

[3]. Manivannan, D., Moni, S. S., & Zeadally, S. (2020). Secure authentication and privacy-preserving techniques in Vehicular Ad-hoc NETworks (VANETs). Vehicular Communications, 25, 100247.
[4]. Alkady, Y., & Rizk, R. (2022, April). Location privacy-preserving of vehicular ad-hoc network in smart cities. In International Conference on Advanced Machine Learning Technologies and Applications (pp. 532-543). Cham: Springer International Publishing.
[5]. Li, H., Pei, L., Liao, D., Sun, G., & Xu, D. (2019). Blockchain meets VANET: An architecture for identity and location privacy protection in VANET. Peer-to-Peer Networking and Applications, 12, 1178-1193.
[6]. Alharthi, A., Ni, Q., & Jiang, R. (2021). A privacy-preservation framework based on biometrics blockchain (BBC) to prevent attacks in VANET. Ieee Access, 9, 87299-87309.
[7]. Ravi, N., Verma, S., Jhanjhi, N. Z., & Talib, M. N. (2021, August). Securing vanet using blockchain technology. In Journal of Physics: Conference Series (Vol. 1979, No. 1, p. 012035). IOP Publishing.
[8]. Ahmed, W., Di, W., & Mukathe, D. (2022). Privacy-preserving blockchain-based authentication and trust management in VANETs. IET Networks, 11(3-4), 89-111.
[9]. Li, H., Pei, L., Liao, D., Sun, G., & Xu, D. (2019). Blockchain meets VANET: An architecture for identity and location privacy protection in VANET. Peer-to-Peer Networking and Applications, 12, 1178-1193.
[10]. Manivannan, D., Moni, S. S., & Zeadally, S. (2020). Secure authentication and privacy-preserving techniques in Vehicular Ad-hoc NETworks (VANETs). Vehicular Communications, 25, 100247.
[11]. Moussaoui, D., Kadri, B., Feham, M., & Bensaber, B. A. (2021, February). A Distributed Blockchain Based PKI (BCPKI) architecture to enhance privacy in VANET. In 2020 2nd international workshop on human-centric smart environments for health and well-being (IHSH) (pp. 75-79). IEEE.
[12]. Lalem, F., Laouid, A., Kara, M., Al-Khalidi, M., & Eleyan, A. (2023). A novel digital signature scheme for advanced asymmetric encryption techniques. Applied Sciences, 13(8), 5172.
[13]. Wang, L., Lin, X., Qu, L., & Ma, C. (2020, June). Ring selection for ring signature-based privacy protection in VANETs. In ICC 2020-2020 IEEE International Conference on Communications (ICC) (pp. 1-6). IEEE.
[14]. Kang, J., Yu, R., Huang, X., & Zhang, Y. (2017). Privacy-preserved pseudonym scheme for fog computing supported internet of vehicles. IEEE Transactions on Intelligent Transportation Systems, 19(8), 2627-2637.
[15]. Memon, I., Chen, L., Arain, Q. A., Memon, H., & Chen, G. (2018). Pseudonym changing strategy with multiple mix zones for trajectory privacy protection in road networks. International journal of communication systems, 31(1), e3437.
[16]. Joy, J., & Gerla, M. (2017, July). Internet of vehicles and autonomous connected car-privacy and security issues. In 2017 26th International Conference on Computer Communication and Networks (ICCCN) (pp. 1-9). IEEE.
[17]. Dorri, A., Kanhere, S. S., & Jurdak, R. (2017, April). Towards an optimized blockchain for IoT. In Proceedings of the second international conference on Internet-of-Things design and implementation (pp. 173-178).
[18]. Lei, A., Cruickshank, H., Cao, Y., Asuquo, P., Ogah, C. P. A., & Sun, Z. (2017). Blockchain-based dynamic key management for heterogeneous intelligent transportation systems. IEEE Internet of Things Journal, 4(6), 1832-1843.
[19]. Amoretti, M., Brambilla, G., Medioli, F., & Zanichelli, F. (2018, July). Blockchain-based proof of location. In 2018 IEEE International Conference on Software Quality, Reliability and Security Companion (QRS-C) (pp. 146-153). IEEE.
[20]. Payattukalanirappel, S. J., Vamattathil, P. V., & Cheeramthodika, M. Z. C. (2024). A Blockchain-Assisted Lightweight Privacy Preserving Authentication Protocol for Peer-to-Peer Communication in Vehicular Ad-hoc Network.
[21]. Shen, H., Wang, T., Chen, J., Tao, Y., & Chen, F. (2024). Blockchain-Based Batch Authentication Scheme for Internet of Vehicles. IEEE Transactions on Vehicular Technology.
[22]. Tandon, R., Verma, A., & Gupta, P. K. (2024). D-BLAC: A dual blockchain-based decentralized architecture for authentication and communication in VANET. Expert Systems with Applications, 237, 121461.
[23]. OPNET, "https://opnetprojects.com/opnet-network-simulator/", Accessed on March 13, 2024.

[24]. VEINS Vehicle in network simulation, “https://veins.car2x.org/”, Accessed on March 18, 2024.
