# Supplementary material for: Blockchain enabled privacy provisioning scheme for location based services in VANETs
Source: PLoS One. 2025 Jun 3;20(6):e0323438. doi: 10.1371/journal.pone.0323438 (PMC12132977; doi:10.1371/journal.pone.0323438)
Supplement: Attachment — Submitted filename: response to reviewers.docx [file pone.0323438.s002.docx]

**Reviewer 1**

**General Comments:** The authors introduce blockchain-based a novel privacy provisioning scheme to secure VANET communication system. Leveraging the privacy and efficiency attributes in anonymizer server, the authors use cache based anonymizer server configured between On-Board Unit (OBU) and Road side unit (RSU) that improve system privacy as well as efficiency. Through performance evaluation, the scheme not only satisfies the aforementioned security and privacy criteria but also proves resilient against various types of attacks, including replay, impersonation, modification, and man-in-the-middle attacks. The paper is well written, but the related work is relatively weak, need to supplement the following literature.

**Author Response:** Thank you very much respected reviewer for the appreciation and encouraging comments.

**Comment 1:** (1) ppru: a privacy-preserving reputation updating scheme for cloud-assisted vehicular networks, (2) tcemd: a trust cascading-based emergency message dissemination model in vanets, (3) trove: a context-awareness trust model for vanets using reinforcement learning. In addition, the abstract of the paper needs further condensed innovation. Paper writing needs to be further strengthened, including typos, chart quality and so on. More details should be provided in the experiment section to ensure reproducibility of the results.

**Author Response:** We are grateful to you for suggesting these studies relevant to our research. We have included the suggested papers in literature review section, and compared the results by adding a comparative analysis of our solution with existing studies in results section.

Regarding the typo or grammatical mistakes, the paper is thoroughly reviewed by English native speaker Prof. Lie Ostervile (Professor of Big Data from USA) and corrected where required. In terms of experimental section for reproducibility of results, we have included additional information such as configuration table 1 defining the necessary attributes for experimental setup,

**----------------------------------------------------------------------------------------------------------------**

**Reviewer 2**

**Comment 1:** The document lacks clarity is some technical definitions, in the use of acronyms and- most importantly- in the proposed blockchain technology protocols and solutions. It is not quite clear why the need to use blockchain since the solution can be accomplished via normal cryptographic applications.

**Author Response:** Respected reviewer, thank you for your valuable feedback. We acknowledge the need for improved clarity in defining technical terms, acronyms, and the specific blockchain protocols employed in our proposed solution. To address this, we have refined our explanations and ensured that all acronyms and terminologies are clearly defined in the revised manuscript.

Regarding the necessity of blockchain, traditional cryptographic mechanisms can offer security, but they often rely on centralized authorities for key management and authentication, which introduces single points of failure and potential bottlenecks. Our blockchain-based approach eliminates these vulnerabilities by leveraging decentralization, immutability, and smart contract automation to enhance security, privacy, and scalability. Specifically, smart contracts facilitate trustless authentication and automated revocation, reducing the need for third-party verification, while blockchain consensus mechanisms prevent malicious modifications to stored data. Furthermore, our framework integrates a cache-based anonymizer server within the blockchain ecosystem, ensuring efficient data handling while maintaining privacy. These features collectively enhance data integrity, resistance to replay attacks, and identity protection, which would be challenging to achieve with conventional cryptographic methods alone.

**----------------------------------------------------------------------------------------------------------------**

**Reviewer 3**

**Comment # 1:** In the abstract, it seems that the only research gap that this work sought to bridge was unobservability. You need to identify additional research gaps and explain how you bridge these gaps in your blockchain-based approach.

**Author Response:** Thank you very much for your keen observation. We have revised the abstract in updated manuscript with research gaps additional such as traffic analysis resistance, unlinkability, and computational efficiency while explaining how the proposed blockchain-based approach addresses them.

**Comment # 2:** Add a summary of performance evaluation in the abstract. This should preferably be in terms of percentage improvements attained against some baseline schemes.

**Author Response:** Thank you very much for your constructive suggestion. We have added improvements in digits as compared to baseline schemes such as “Performance evaluations demonstrate that our scheme significantly reduces computational costs, achieving 0.23972 for V2V communication and 0.13166 for V2RSU communication outperforming referenced schemes. Additionally, our approach reduces communication costs to 530 bits for V2V and V2RSU, compared to 1664-4416 bits in existing methods. The cache hit ratio improves system responsiveness, and our model achieves a location leakage probability of just 0.05%, significantly lower than centralized architectures. Furthermore, our scheme ensures strong privacy protection, attaining a maximum entropy level of 5, outperforming existing solutions.”

**Comment # 3:** Your first contribution reads as follows:

"To overcome the challenges in centralized systems, this study introduces blockchain technology and devises an innovative decentralized framework known as the blockchain-based VANET. Proposed approach not only guarantees the integrity and security of SBMs but also conserves storage space and reduces processing time within the blockchain."

--- It is not clear how your proposed solution conserves storage space and reduces processing time within the blockchain. What specific technique have you deployed to achieve these two performance gains?.

**Author Response:** Thank you for your valuable comment.

**Regarding Processing Time Reduction:**

Our authentication scheme leverages a lightweight cryptographic mechanism, reducing the computational burden on OBUs and RSUs. As seen in our results, our scheme achieves 0.23972 for V2V and 0.13166 for V2RSU in computational cost, significantly outperforming referenced methods.

**Regarding Reduced Blockchain Overhead via Batch Processing:**

Instead of validating each authentication request individually, we deploy a batch processing approach using Merkle tree-based validation. This allows multiple authentication requests to be processed together, reducing the number of cryptographic operations and speeding up validation times.

**About Efficient Key Management with Short-Lived Pseudonyms:**

By utilizing ephemeral pseudonyms that are dynamically refreshed, we reduce the frequency of blockchain transactions related to key updates. This decreases both storage and processing load.

**Comment # 4:** Improve the clarity of your third contribution.

**Author Response:** Thank you for your kind comment, and we do agree that it

**Comment # 5:** Give the summary of the results of the evaluation in your fourth contribution.

**Author Response:** We acknowledge the reviewer’s suggestion to update the contribution statement. We have added more details such as “Our proposed scheme reduces computation cost (0.23972 for V2V, 0.13166 for V2RSU) and communication cost (534 and 530 bits for V2V, 530 bits for V2RSU), outperforming existing methods. The cache-based anonymizer server enhances efficiency with a high cache hit ratio, while our privacy measures lower location leakage probability to 0.05% and achieves a maximum entropy level of 5, ensuring strong security and privacy in VANET systems.”

**Comment # 6:** The Related works in section 2 is not well done. Instead of delving into detailed descriptions of the presented works, pay particular attention to the technology deployed, strengths and weaknesses. This will help build the research gaps. At the end of Section 2, add a table to compare and contrast all the related works.

**Author Response:** We really appreciate the reviewer’s keep observation. We have updated our literature review section by adding three more recent studies relevant to the proposed framework. Also we have summarized the existing studies and presented with comparative analysis in table 2.

**Comment # 7:** Algorithm 1 is poorly structured and fails to use conventional notations for decision points, as well as looping. The same applies to Algorithm 2 and Algorithm 3.

**Author Response:** Thank you and we do acknowledge your valuable comment. We have revised all the algorithms accordingly in updated manuscript.

**Comment # 10:** The Security Analysis in Section 5 is too brief and fails to account for majority of the typical VANET threats. In addition, you need to add formal security verification. Moreover, you need to specify the attack/threat model against which the security analysis is being conducted.

**Author Response:** Thank you very much for your keen observation. Actually, we have addressed the concerns including “A. Query content modification attack resistance, B. Content Replay attack resistance, C. Impersonation attack resistance, D. Middle man attack resistance”. Accordingly, we have given the theoretical security analysis model and description.

**Comment # 11:** Give the units for 0.23972. Is this minutes/seconds/ milliseconds?

**Author Response:** Thank you for your keen observation. It is in milliseconds (ms). We have updated the manuscript by mentioning the units accordingly.

**Comment # 12** You claim that your scheme demonstrates notably lower communication costs, with 534 and 530 bits compared to 1664, 2912, and 2560 bits for references [12], [21], and [22], respectively.

**Author Response:** Thank you for your comment. To clarify, the communication cost values of 534 and 530 bits for our scheme, compared to 1664, 2912, and 2560 bits for the referenced schemes [12], [21], and [22], respectively, reflect the efficiency gains achieved by our proposed solution. These reductions are primarily due to the integration of cache-based anonymizer servers, which minimize the need for repeated data transmission and reduce overall message overhead. This significant reduction in communication costs underscores the effectiveness of our approach in optimizing resource utilization within VANET systems.

**Comment # 16:** It is not clear how you arrive at 534 and 530 bits.

**Author Response:** Thank you for your valuable comment. The values of 534 and 530 bits for communication cost were derived from a detailed analysis of the data transmission overhead in our proposed scheme. Specifically, these figures represent the average communication cost for V2V and V2RSU communications, calculated by evaluating the bandwidth consumption and transmission time across multiple communication links in the system. We accounted for factors such as message size, encryption overhead, and the caching mechanism employed by the anonymizer server, which collectively contribute to the reduction in communication cost. These values were validated through experiments in the OPNET and Veins vehicular network simulation frameworks.

**Comment # 17:** In Section 7, describe some of the practical implications of your findings as well as possible limitations of your approach.

**Author Response:** Thank you for your comment. The practical implications of our findings suggest that the proposed privacy provisioning scheme can significantly enhance the efficiency and security of VANET systems, particularly in real-time applications such as intelligent transportation and vehicular safety systems. By reducing communication and computation costs, as well as improving data privacy, our approach could be deployed in urban traffic networks to safeguard sensitive vehicle data while optimizing resource utilization. However, some limitations of our approach include its reliance on a cache-based anonymizer server, which may introduce challenges in highly dynamic environments with frequent topology changes. Additionally, while the system demonstrates strong resilience to common attacks, further research is needed to evaluate its performance in large-scale, real-world VANET deployments.
