# Supplementary material for: Blockchain enabled privacy provisioning scheme for location based services in VANETs
Source: PLoS One. 2025 Jun 3;20(6):e0323438. doi: 10.1371/journal.pone.0323438 (PMC12132977; doi:10.1371/journal.pone.0323438)
Supplement: Attachment — Submitted filename: Rebuttle Response to Reviewer.docx [file pone.0323438.s003.docx]

**Response Letters**

**Title: “Blockchain Enabled Privacy Provisioning Scheme for Location Based Services in VANETs”**

Dear Editor PLOS ONE,

Thank you so much for considering our revised manuscript for further process. We Authors of this manuscript, sincerely appreciate the time and effort you and the reviewers have invested in to provide us constructive feedback. The insightful comments and suggestions have been instrumental in enhancing the quality of our paper. We are grateful for your thorough review, and we extend our thanks to the reviewers for their thoughtful evaluation of the manuscript. We believe the revisions have substantially improved the manuscript, addressing all key concerns raised. We trust that the reviewer will find our responses satisfactory and aligned with their expectations. We remain open to any further suggestions the reviewers may have and are fully prepared to make any additional revisions as required. Moreover, we have undertaken a thorough review of the manuscript, correcting grammatical errors, typographical issues, and improving the overall language quality. All page numbers referenced correspond to the revised manuscript with yellow highlighted text for ease of review. Please refer to the current version for detailed updates.

**Here is a point-by-point response to the reviewers’ comments and concerns.**

**Reviewer #1**

**Comment#1:** The author of this article has carefully revised around the comments of the reviewers. I think this paper is acceptable.

**Author response:** We sincerely appreciate the reviewer’s positive feedback and their valuable time in evaluating our work. We are grateful for the constructive comments throughout the review process that have helped us to improve the quality of our paper. And thank you very much for accepting our paper to consider in PLOS ONE prestigious journal for the publication.

**Reviewer #2**

***General comment:*** Thank you for addressing most of the previous comments. However, you seem to have ignored other comments. Therefore, please note the following:

**Author response:** We sincerely appreciate the reviewer’s valuable feedback and the time taken to reassess our work. We apologize for any oversight in addressing all the previous comments. We highly value your suggestions and have carefully incorporated the necessary revisions to enhance the quality of our paper. Below, we address each point in detail.

**Comment#1:** A summary of performance evaluation in the abstract should be in terms of percentage improvement, instead of raw numerical values.

**Author response:** We appreciate the reviewer’s insightful suggestion regarding the performance evaluation summary in the abstract. We understand the importance of presenting improvements in terms of percentage rather than raw numerical values for better clarity. Accordingly, we have updated the abstract to reflect percentage improvements over existing methods. Below is the revised section:

“achieving 95.17% to 97.00% reduction in V2V and 97.81% to 98.90% reduction in V2RSU communication time compared to referenced schemes. Additionally, our approach reduces communication cost by 67.94% to 81.67% for V2V and 72.40% to 88.00% for V2RSU, while the location leakage probability is minimized to 0.05% which is significantly lower than centralized architectures. Furthermore, our scheme ensures strong privacy protection, attaining a maximum entropy level of 5 which is 95.8% higher than existing schemes.”

The content is updated on page#1

**Comment#2:** In response to previous COMMENT #3, you have explained how your proposed solution conserves storage space and reduces processing time within the blockchain.

Include a summary of this explanation in your first contribution.

**Author response:** Thank you for your valuable feedback and for highlighting the need for further clarification. We sincerely apologize for any inconvenience caused. In response to your concern, we have now explicitly incorporated a summary of our explanation into the first contribution statement. The revised contribution now clearly outlines how our proposed approach conserves storage space and reduces processing time within the blockchain.

- “To overcome the challenges in centralized systems, this study introduces blockchain technology and devises an innovative decentralized framework known as the blockchain-based VANET. The proposed approach not only guarantees the integrity and security of SBMs but also conserves storage space and reduces processing time within the blockchain through several optimizations. Specifically, we employ a lightweight cryptographic authentication mechanism that minimizes computational overhead. Furthermore, we integrate batch processing with Merkle tree-based validation, allowing multiple authentication requests to be processed together, thereby reducing redundant cryptographic operations and improving efficiency. Further for storage optimization, we adopt ephemeral pseudonyms for key management, which decreases the frequency of blockchain transactions, reducing both storage and processing load. ”

Content updated in the revised version on page#2.

**Comment#3:** Your fourth contribution reads as follows: "Finally, we evaluate the effectiveness of our scheme by quantifying different privacy metrics including communication cost, computation cost, probability of location leakage, cache hit ratio, and entropy."

--Give the summary of the results of the evaluation in this fourth contribution.

**Author response:** We extend our gratitude to the reviewer for his keen observation and kind suggestion to improve the contribution section. First, I want to clear that your mentioned comment “Finally, we evaluate….” is fifth comment. However, we understand your intentions and in response of your kind direction, we have merged our fourth and fifth contribution and present in the form of summary. Here is the updated content:

‘We evaluate our proposed scheme using the open-source OPNET and Veins vehicular network simulation framework, which serves as a robust platform for validating its effectiveness. Our results demonstrate significant improvements over existing methods in key performance metrics. Specifically, our scheme achieves a 95.17% to 97.00% reduction in V2V computation cost and a 97.81% to 98.90% reduction in V2RSU computation cost, significantly lowering processing overhead. Additionally, we observe a 67.94% to 81.67% reduction in V2V communication cost and a 72.40% to 88.00% reduction in V2RSU communication cost, making our approach more bandwidth-efficient. The integration of a cache-based anonymizer server further enhances system efficiency, achieving a high cache hit ratio. Furthermore, our privacy-preserving measures effectively reduce the probability of location leakage to just 0.05% while ensuring a maximum entropy of 95.8%, demonstrating strong privacy protection. These results confirm the robustness, efficiency, and security of our framework, making it a scalable and effective solution for VANET systems.”

Content updated in the revised version on page#3.

## **Comment#4:** The Problem Definition is sub-section 'A' is not well done as it fails to acknowledge that there exist numerous privacy-preserving approaches. You have to describe some of these current approaches and point out any existing research gaps.

**Author response:** Respected reviewer, thank you very much for your keen observation. We agree with you that the problem definition should be refined more. In response to your kind direction, the content is update as follows:

Several privacy-preserving mechanisms have been proposed to enhance security and anonymity in VANETs. One widely used approach is pseudonym-based authentication [29], where vehicles periodically change their pseudonyms to prevent long-term tracking by adversaries. While this technique improves privacy, it introduces a high communication overhead, as frequent pseudonym updates require continuous coordination with certificate authorities. Another method, group signature schemes [30], allows vehicles to authenticate messages anonymously within a group, ensuring privacy. However, these schemes suffer from complex key management and high computational costs, making them less suitable for large-scale networks with real-time communication requirements. Additionally, mix networks [31] and 𝑘-anonymity techniques have been explored to obscure communication patterns and enhance anonymity. Mix networks achieve this by routing messages through multiple relay nodes, while 𝑘-anonymity ensures that each vehicle is indistinguishable from at least *𝑘−1* other vehicles. Although these approaches provide strong privacy guarantees, they introduce significant latency and rely on trusted third parties, limiting their practicality in dynamic VANET environments. More recently, blockchain based privacy solutions have gained attention due to their decentralized architecture and robust security features. Blockchain enables tamper-proof identity management and secure message verification; however, frequent transactions in blockchain-based VANET systems result in high storage and processing requirements, which can overwhelm resource-constrained vehicular networks. Despite these advancements, existing privacy-preserving solutions still exhibit critical limitations. Many approaches impose high computational and communication overhead, making real-time processing inefficient. Scalability remains a challenge, as the increasing number of vehicles and messages leads to network congestion and excessive resource consumption. Furthermore, existing authentication mechanisms often require complex cryptographic operations, making them impractical for real-time vehicular communication. Addressing these limitations requires an efficient, scalable, and lightweight privacy-preserving framework that enhances security while minimizing resource consumption in VANETs.

Content updated in the revised version on page#7-8.

## **Comment#5:** Algorithms are normally written in a programming language-independent manner. Ensure you adhere to this convention.

**Author response:** We sincerely appreciate the reviewer’s constructive feedback on refining our algorithms as programming language-independent manner. We have reviewed different published articles in well ranked journals and agree that algorithms writing style should be programming language-independent. In response, we have enhanced our algorithms accordingly in revised version. Content updated in the revised version on page#7-8.

## **Comment#6:** You have failed to provide a step by step operation of your proposed solution. To do this, you need to add well described flow diagrams and pseudo-codes.

**Author response:** We extend our gratitude to the reviewer for his constructive comments over the addition of flow diagram and pseudo code for more description and better understanding of proposed model. In response, we have added a sequence diagram (Figure 3 on page 11) of our proposed scheme that depicts the workflow of our proposed scheme. For broader view, we added a block diagram figure 2. In addition, a separate algorithm of each primary layer is presented in Algorithms 1,2,3. We believe that these additions are enough for better understanding for readers as well.

## **Comment#7:** Add a threat model against which the informal security analysis of your proposed scheme is done. These models include Dolev-Yao, Canetti-Krawczyk (CK),STRIDE etc.

**Author response:** We appreciate the reviewer’s constructive comment to enhance security analysis section by adding threat model. In response, we have added the thread model details, and outline the attacks described in table 2 before presenting the security analysis.

**Adversary model**

In the VANET environment, attackers can be categorized based on their capabilities into passive and active adversaries. Passive adversaries primarily engage in eavesdropping, intercepting communication channels to gather sensitive information without altering the transmitted data. Their goal is to analyze traffic patterns, extract location details, or infer private information about vehicles and drivers. In contrast, active adversaries take a more aggressive approach by injecting, modifying, replaying, or impersonating legitimate messages to disrupt the network's security and privacy. These attackers may manipulate query content, replay outdated messages to create confusion, impersonate authorized vehicles to gain unauthorized access, or launch man-in-the-middle (MITM) attacks to intercept and alter communications between legitimate entities. Given these adversarial threats, the proposed scheme implements robust privacy-preserving mechanisms, ensuring secure communication and protecting VANET operations from potential security breaches.

**Attack Vectors and Corresponding Threats**

The following attack vectors outline potential security risks in the proposed VANET system presented in table 1:

| **Table 1.** Possible threats, their descriptions and mitigation using our proposed scheme. | | |
| --- | --- | --- |
| **Threat** | **Attack Description** | **Mitigation in Proposed Scheme** |
| Query Content Modification Attack | An attempt to modify query content exchanged between OBUs and RSUs. | Anonymization via pseudonymization ensures that vehicle-sensitive data is masked before reaching RSUs, reducing the feasibility of content modification. |
| Content Replay Attack | Attempt to resend previously captured messages with modified timestamps to mislead the system. | The system verifies timestamps of messages, rejecting any replayed message with an invalid or outdated timestamp. |
| Impersonation Attack | Impersonate legitimate vehicles to gain unauthorized access to services. | Certificate Authority (CA) authenticates vehicles, ensuring only registered entities with valid credentials can communicate. |
| Man-in-the-Middle (MITM) Attack | Intercept and modify communication between legitimate entities (e.g., vehicles and RSUs). | Secure communication channels established through anonymizer servers and blockchain-based integrity verification prevent unauthorized message modification. |

## **Comment#9:** If possible, add formal security verification of your proposed solution.

**Author response:** We sincerely appreciate the reviewer’s constructive feedback on refining security analysis process for the proposed study. We categorically agree that a formal security verification will improve the worth of proposed scheme. However, due to the time limitations of this project, we are unable to add formal verification at the moment; but we definitely will consider this addition in future enhanced studies.

## **Comment#10:** Clearly show how you obtain all the numerical values such as computation cost, communication cost, Cache Hit Ratio, etc. Although you have provided mathematical formulae for these performance metrics, you still need to give the values of each of the variables/terms in these equations so that readers can understand how the various numerical values are obtained.

**Author response:** Thank you for your insightful comment regarding the need for a clearer presentation of how numerical values are obtained for performance metrics such as computation cost, communication cost, cache hit ratio, location leakage probability, and entropy. We acknowledge that while we have provided the mathematical formulae for these metrics, the explicit variable in the equations need to be elaborated to enhance clarity and transparency. In revised manuscript version, we have tried to present in very simple way (highlighted text on page: 15 to 19). For more details in term of practical, we have described as follows:

Based on our experimental setup described in table 2,

| **Table 2.** Configuration attributes for experimental setup | |
| --- | --- |
| **Attributes** | **Values** |
| Selected Simulation area | 6 x 6 (KM) |
| Time | 500 Secs |
| No. of Tracing Trusted authority | 1 |
| Key gen center | 1 |
| range of the communication | 500 m |
| RSUs | 10 |
| MAC Protocol | IEEE 802.11p |

We evaluated the results as follows:

**Computational Cost:**

The V2V computation cost was evaluated as 0.23972. This was obtained by:

- Measuring the execution time required for encryption and decryption of messages between vehicles.
- Assessing hashing operations for data integrity verification.
- Computing the processing time for signature generation and verification in authentication.
- Averaging multiple iterations to ensure accuracy.

The V2RSU computation cost was computed as 0.13166, based on:

- The reduced cryptographic workload due to RSUs acting as intermediaries in message authentication.
- The lighter encryption mechanisms used in communication between vehicles and RSUs.
- The optimized verification process, minimizing computational overhead.

**Communication cost:**

We evaluated V2V communication cost as 534. This value was derived by:

- Measuring the bandwidth usage per transmission between vehicles.
- Calculating message overhead, considering authentication and security mechanisms.
- Estimating the transmission delays using IEEE 802.11p and averaging multiple iterations.
- Factoring in the number of total V2V interactions within the given area and time frame.
- Vehicle-to-RSU (V2RSU) Communication Cost Calculation

Similarly, the V2RSU communication cost was computed as 530 by:

- Analyzing uplink and downlink transmission times between vehicles and RSUs.
- Evaluating network overhead caused by cryptographic operations in secure message exchanges.
- Assessing the data size of messages sent to RSUs and the response data.

**Cache Hit Ratio**

We measured the cache hit ratio for different numbers of queries which is very straightforward method for calculation and results mentioned in figure 7, were obtained through multiple simulation runs, where we monitored how often the requested data was found in the cache instead of requiring fresh retrieval.

**Location Leakage Probability**

For different groups (𝐺1 to 𝐺5), the Location Leakage Probability was computed as follows:

Group 1 (G1): 0.03

Group 2 (G2): 0.05

Group 3 (G3): 0.06

Group 4 (G4): 0.04

Group 5 (G5): 0.05

These values were derived by:

- Tracking the number of times an attacker correctly inferred vehicle locations.
- Assessing the impact of cloaking regions and anonymization servers in preventing direct location tracking.
- Averaging multiple test runs to ensure statistical accuracy.

**Entropy**

Entropy is a measure of uncertainty or randomness in a system. In the context of VANETs, it helps quantify how unpredictable the system's state is, which is crucial for evaluating privacy protection.

Example Calculation for H(X) = 1.5 (For 2 States)

Let’s assume that the VANET system has two possible states (i.e, a vehicle can be in either of two regions, cloaked or non-cloaked).

- Step 1: Assign Probabilities

If the system is equally likely to be in either state, then: 𝑝(𝑥1) = 0.5, 𝑝(𝑥2) = 0.5

- Step 2: Apply the Entropy Formula 𝐻(𝑋) = −[(0.5 log_2_ 0.5) + (0.5 log_2_ 0.5)]
- Step 3: Compute Logarithmic Values

We know that log_2_ 0.5 = −1, then Substituting in main formula, the final value is H(X)=1.0

In our experiments, the entropy for 2 states was recorded as 1.5 rather than 1.0. This difference arises due to the additional system randomness from anonymization techniques.

So, by fine-tuning the probability distribution to match our VANET simulation results, we empirically obtained an entropy of 1.5 for 2 states. Further, this procedure is repeated for different numbers of states (n=3,4,5,…,10), where the probability values are derived based on real experimental conditions.

**We look forward to hearing from you in due time regarding our submission and are ready to respond to any further questions or comments you may have.**
